# Integra: An Alternative Option for Reconstruction of Extensive Finger Defects With Exposed Bones

**DOI:** 10.7759/cureus.16223

**Published:** 2021-07-07

**Authors:** Ahmed Mustafa Hamdan, Muath Mamdouh Mahmod Al-Chalabi, Wan Azman Wan Sulaiman

**Affiliations:** 1 Reconstructive Sciences Unit, Universiti Sains Malaysia (USM), Kota Bharu, MYS; 2 Plastic and Reconstructive Surgery, Universiti Sains Malaysia (USM) School of Medical Sciences, Kota Bharu, MYS

**Keywords:** integra, finger trauma, finger reconstruction, exposed phalangeal bones, exposed tendons

## Abstract

Extensive soft tissue injuries with exposed joints, bones, and tendons due to trauma of the hand -particularly fingers- necessitate soft tissue reconstruction and coverage. However, these injuries are challenging; therefore, various management options for traumatic skin loss over fingers are widely performed. These options comprise wound care permitting wound contracture without surgical intervention, full or split-thickness skin grafting, skeletal shortening and primary closure, and various types of flaps. We present a case of successfully placed Integra over the exposed phalangeal bones followed by split-thickness skin grafting a few weeks later, with a good outcome. We conclude that Integra is an alternative, safe and effective method for reconstructing severely traumatized fingers with exposed bone, tendon, or joint without sacrificing outcome.

## Introduction

Reconstructive surgeons face a challenging task when dealing with complex wounds involving the hand. Due to the scarcity of overlying soft tissue and the composite anatomical structure of the hand, trauma and surgical injuries often result in denuded vital structures, such as tendons, bones, and joints. In addition, because of the inability to perform primary closure of the wound, these vital structures are frequently exposed and lack adequate vascularized overlaying soft tissue, leaving them prone to dryness and infection. Furthermore, exposed cartilage, tendon, or bone lack enough vascularity to support wound bed granulation for re-epithelialization or new vascular formation, necessary for skin graft take and survival [1]. These reconstructive problems lead to frequent use of various flaps, including local, regional, and micro-vascular free flaps, to close wounds of the hands.

Although flaps are considered an excellent option for the reconstruction of hand defects, they have some disadvantages. These include the risk of flap loss and donor site morbidity, as well as, the bulkiness of the flap, experience, and microsurgical capability in the case of free flaps. 

Integra may offer an option for immediate coverage of these vital structures in one's hand (i.e., bones, tendons, and joints). Integra is “a bilaminar dermal regeneration template, composed of a silicone protective sheet overlying a matrix composed of bovine collagen and glycosaminoglycans derived from shark chondroitin-6-sulfate” [2]. We present our clinical experience with a patient who successfully underwent Integra placement over the exposed phalangeal bones, followed by split-thickness skin grafting a few weeks later.

## Case presentation

**Figure 1:**
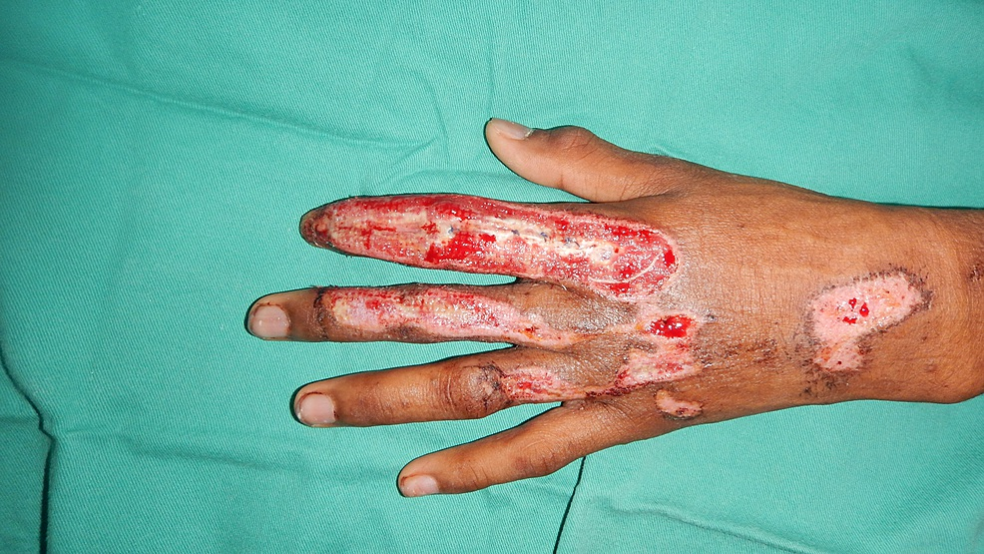
Laceration wounds over the index, middle, and ring fingers.

A 13-year-old boy was a victim of a motorbike accident. He lost control of his motorbike when a giant lizard crossed the road and fell on the ground on his left side with his left hand grazed on the ground. He sustained a de-gloving wound over the dorsum aspect of the left index finger from the metacarpophalangeal joint till distal interphalangeal joint, partial laceration of the extensor tendon at zone IV with total loss of the tendon at zone I, II, and III, and exposed all phalangeal bones (Figure 1). He could not extend his index finger due to loss of tendon neither could he flex it due to pain. He also sustained a laceration wound over the dorsum of the left middle finger at the level of the middle phalanx with partial laceration of the extensor tendon at zone II (30%). In addition, the left ring finger sustained a laceration wound at the proximal phalanx level over the dorsum, with partial laceration of the extensor tendon at zone III (20%). The patient was able to extend his left middle and ring finger at the metacarpophalangeal, proximal, and distal interphalangeal joints.

**Figure 2:**
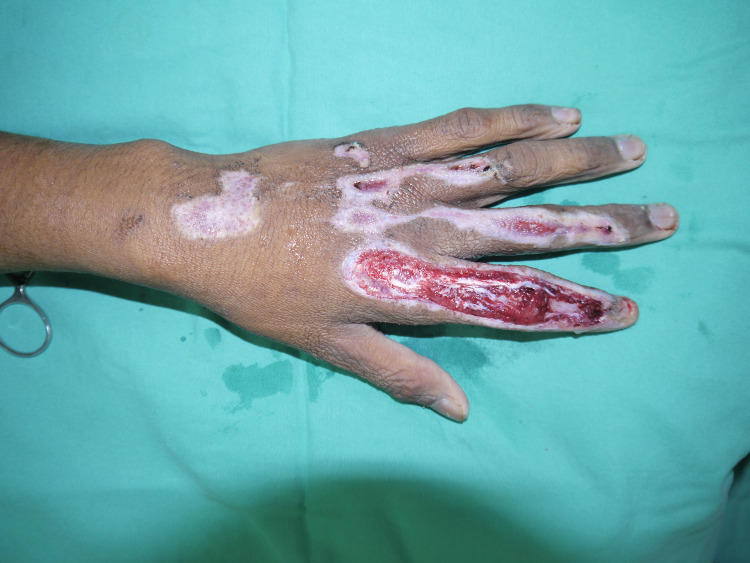
Appearance of the laceration wounds after surgical debridement.

**Figure 3:**
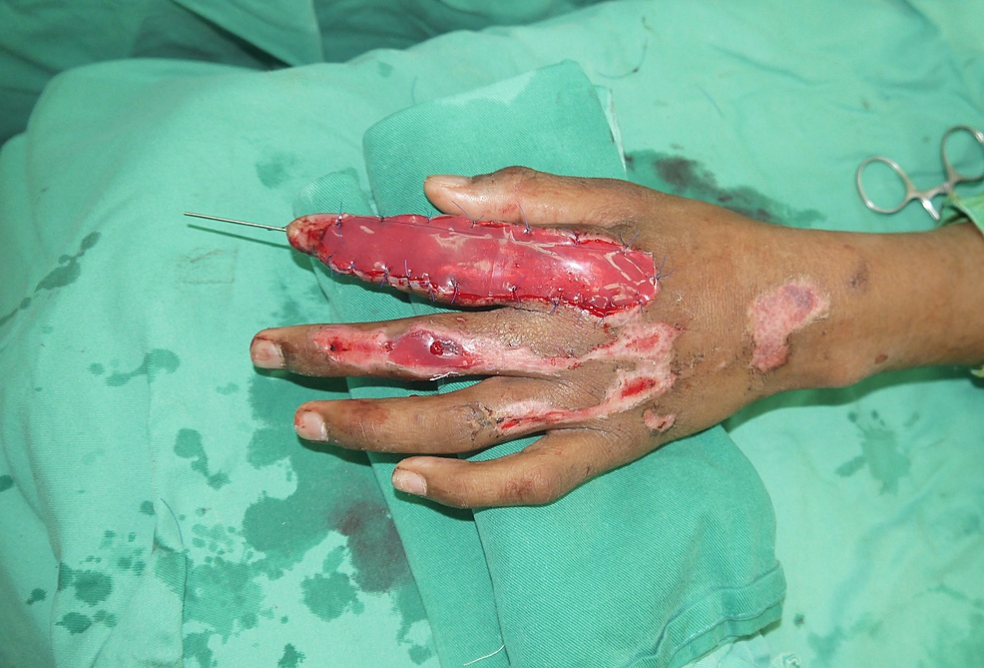
K-wire in the index finger, and Integra appears over the wound of the index and middle fingers.

**Figure 4:**
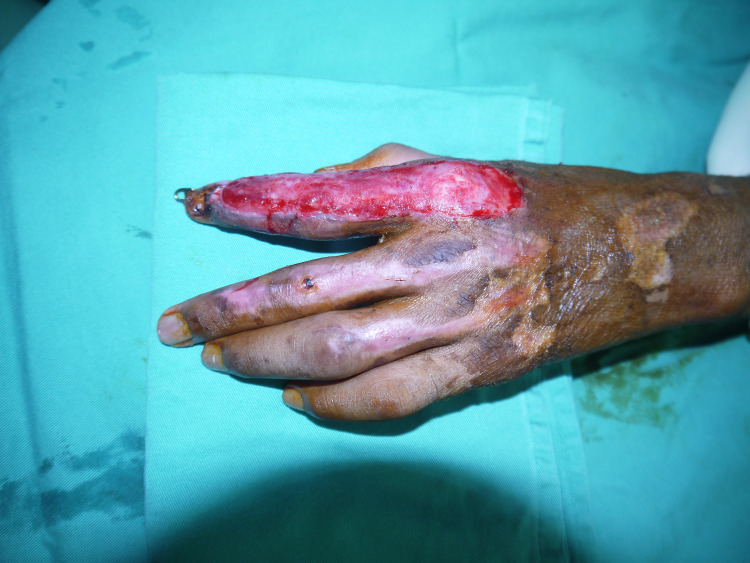
Neo-dermis cover the extensor surface of the index finger after removal of the silicon layer.

Proper wound debridement was performed (Figure 2), and Integra was applied over the wound of the left index and middle fingers and fixed to the skin with nylon 5-0 (Figure 3). Next, the acti-coat dressing was applied over the Integra. On postoperative day three, the first dressing was changed, after which a bi-weekly inspection of the wound was done to evaluate for infection and observe the degree of vascularization of the neo-dermis underneath the silicone layer (Figure 4).

A skin graft (split-thickness) was applied over the left index finger three weeks after the application of Integra. Due to their limited size, the wounds over the left middle and index fingers were treated with dressing changes until successful healing.

The patient was evaluated at follow-ups until the complete healing of the skin graft. In addition, hand therapy was prescribed to achieve maximum mobility for covered soft tissue.

**Figure 5:**
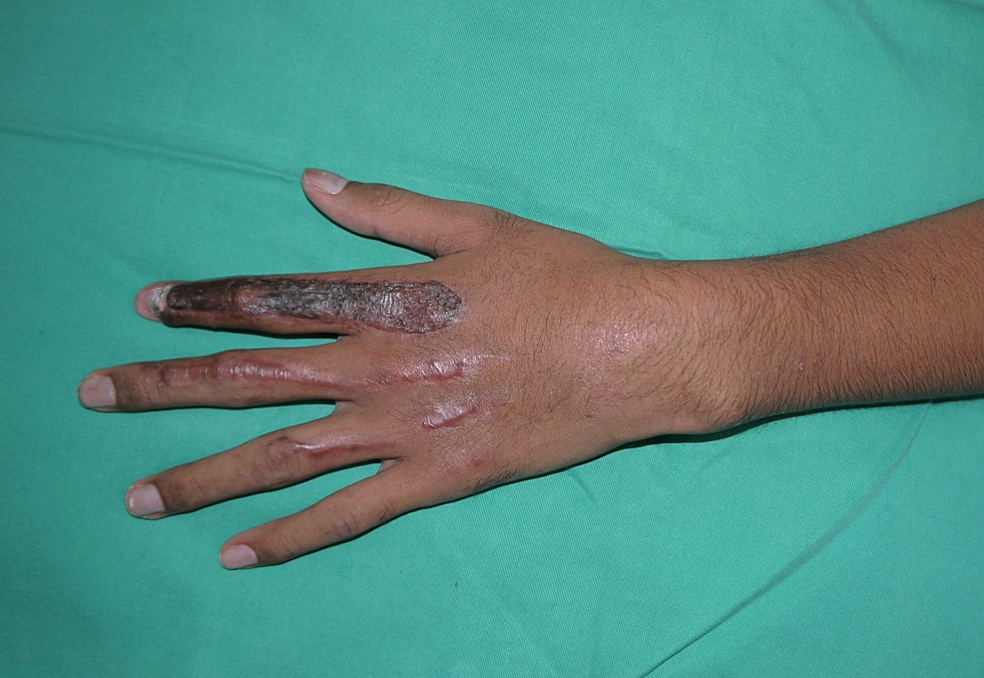
The appearance of split-thickness skin graft over the extensor surface of the index finger after 10 months.

At the 10th month follow-up, the skin was fully healed (Figure 5), and the patient's active distal interphalangeal joint motion was 0 to 50 degrees, proximal interphalangeal joint 0 to 45 degrees, and metacarpophalangeal joint 0 to 90 degrees. Therefore, no further operation for tendon reconstruction was deemed necessary.

## Discussion

Soft tissue injuries with exposed bone and tendons in the hand may present several challenges for coverage. The exposed bone and tendon do not have enough blood supply to provide a bed’s granulation required for fast re-epithelialization or neovascularization. In addition, if exposed bone and related fractures are not properly covered by soft tissue, they face a significant risk of infection and desiccation [1]. These defects pose challenging reconstructive problems because of the restricted range of motion, inadequate cosmetic appearance, tendon adhesions, and donor site morbidity.

When it comes to the reconstructive ladder, skin grafts may not be a viable option in the presence of a non-vascularized wound bed such as exposed bone or tendon with damaged periosteum or paratenon. That leaves the surgeon with local flaps, regional flaps, free flaps, and skin substitutes.

When treating tissue defects in the hand complex, flaps are an excellent option, as they replace like with like, allow minimal scarring and early rehabilitation. Nevertheless, a few general disadvantages are related to flap procedure: risk of flap loss, increase in operating time, restricted movement in some flaps like cross-finger flap and groin flap, donor site morbidity, and a few other disadvantages in the case of free flaps include the bulkiness of these flaps, experience, knowledge, and microsurgical ability. Concerning complications, Khouri and colleagues found that the rate of flap loss was 4.1%, with a 12.1% chance of encountering some measured complications, including arterial insufficiency, wound breakdown, and flap necrosis [3].

Integra may offer an alternative choice for immediate coverage of complex and extensive hand wounds with exposed bones, tendons, and joints. Integra is “a bilaminar dermal regeneration template, composed of a silicone protective sheet overlying a matrix composed of bovine collagen and glycosaminoglycans derived from shark chondroitin-6-sulfate.” The thin silicone sheet on the surface works as an artificial epidermis. The deeper porous layer of cross-linked bovine tendon collagen and chondroitin-6-sulfate permits fibroblasts’ migration through it to regenerate the dermal layer [2]. Three weeks later, epidermal grafting of the newly generated dermis can be performed.

Since it was first described by Burke et al., burn surgeons have been utilizing Integra to produce a neo-dermis in full-thickness burns treatment [4-5]. It has also been proven to repair non-burn-related complex tissue defects effectively [6]. Integra was used to cover hand wounds with severe trauma in 15 patients by Weigert et al., who found that it was successful in 87% of their patients, with good function and cosmetic results [7]. Shores et al. reported a 95.5% take of split-thickness skin grafts (range: 85%-100%) when applied over Integra used to cover exposed tendon with no peritenon in wounds of the upper limbs measuring an average of 30.3 (range: 4-96 cm2) [8]. In a study conducted by Nicholas et al., 14 patients with exposed bones, joints, or tendons had their hands reconstructed with Integra. As a result, 13 patients had effective reconstructions, with a 97% skin graft take rate. Preoperative hand function was restored in 92% of patients. In addition, 85% of patients were extremely satisfied with the cosmetic outcome while 15% were satisfied to a degree [9].

The main downside of using Integra is the need for a second interventional procedure to harvest and place the skin graft. This is usually done two or three weeks following the initial Integra placement. However, a one-stage procedure, followed by secondary intention healing, can be conducted in an outpatient setting, reducing the patient's burden and medical costs [10].

## Conclusions

Integra is considered an efficient and safe method for treating non-burn hand wounds with exposed tendons, bones, and joints. It allows the regeneration of neovascularized tissue over the denuded or exposed structures within these wounds, resulting in a well-vascularized tissue bed for later skin graft application. Thus, it provides an efficient, minimally invasive alternative to more demanding reconstructive procedures that have potential morbidity without sacrificing outcome.
